# Supplementary material for: Tailoring Thermal Conductivity of Single-stranded Carbon-chain Polymers through Atomic Mass Modification
Source: Sci Rep. 2016 Oct 7;6:34999. doi: 10.1038/srep34999 (PMC5054521; doi:10.1038/srep34999)
Supplement: Supplementary Information [file srep34999-s1.pdf]

Supporting Information

Tailoring Thermal Conductivity of Single-stranded Carbon-chain Polymers through Atomic Mass Modification

Quanwen Liao[1], Lingping Zeng[2], Zhichun Liu[1,*], Wei Liu[1,*]

1. School of Energy and Power Engineering, Huazhong University of Science and Technology (HUST), Wuhan 430074, China

2. Department of Mechanical Engineering, Massachusetts Institute of Technology, Cambridge, Massachusetts 02139, USA

* Corresponding authors. E-mail: zcliu@hust.edu.cn (Zhichun Liu), w_liu@hust.edu.cn (Wei Liu)

MD simulations

The thermal conductivities of Model C-X and Model C-H_mY_n are calculated by equilibrium molecular dynamics through the Green-Kubo formula. The thermal conductivity is the mean value of six realizations with different initial conditions. Figure S1-S4 show typical heat flux autocorrelation function and their integrations for Model C-X and Model C-H_mY_n during EMD calculations.

Figure S1 and S2 show the heat flux autocorrelation function and thermal conductivity versus correlation time for Model C-X with m_X = 0.5 g/mol and m_X = 80 g/mol, respectively. Figure S1 shows the HFACF decays to zero in 10ps. The blue curve of thermal conductivity converges within 10ps which is consistent with the decay of HFACF.

Figure S3 and S4 shows the heat flux autocorrelation function and thermal conductivity versus correlation time for Model C-H_mY_n with m_Y = 17 g/mol and Φ_H = 50% and Model C-H_mY_n with m_Y = 35.5 g/mol and Φ_H = 50%, respectively.

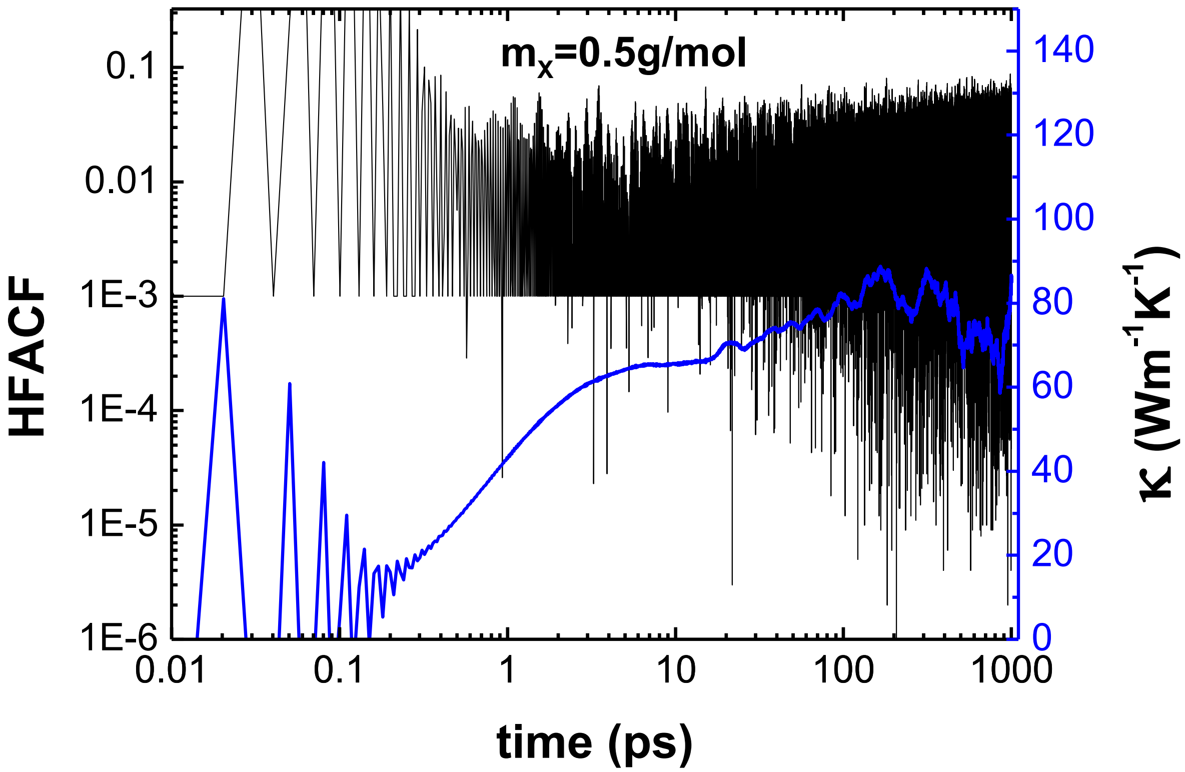

Figure S1. The heat flux autocorrelation function and thermal conductivity versus correlation time for Model C-X with m_X = 0.5 g/mol.

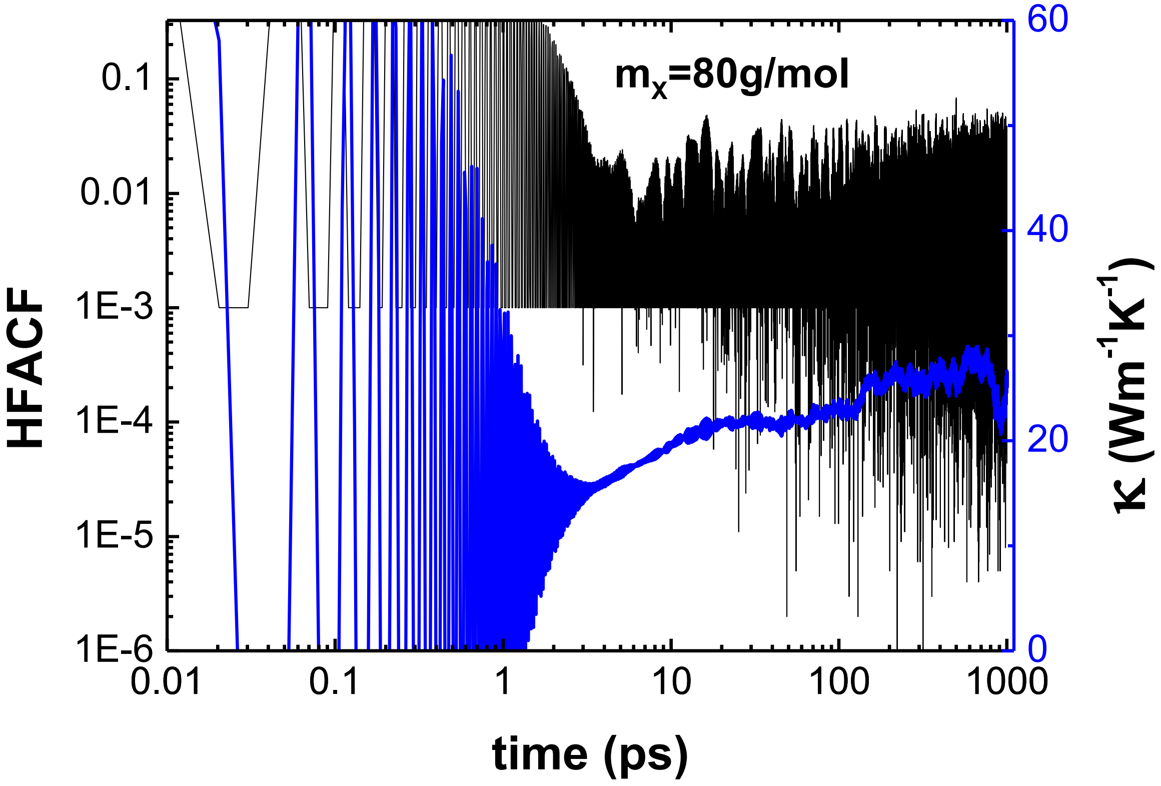

Figure S2. The heat flux autocorrelation function and thermal conductivity versus correlation time for Model C-X with m_X = 80 g/mol.

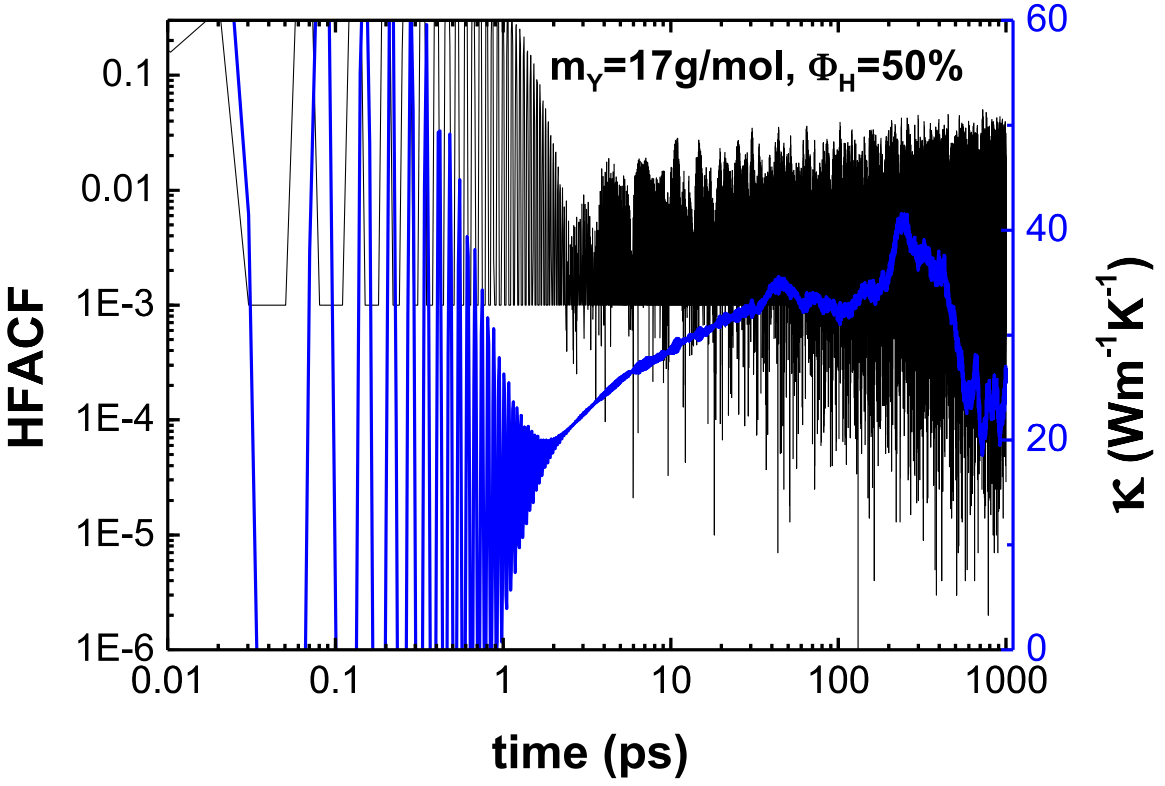

Figure S3. The heat flux autocorrelation function and thermal conductivity versus correlation time for Model C-H_mY_n with m_Y = 17 g/mol and Φ_H = 50%.

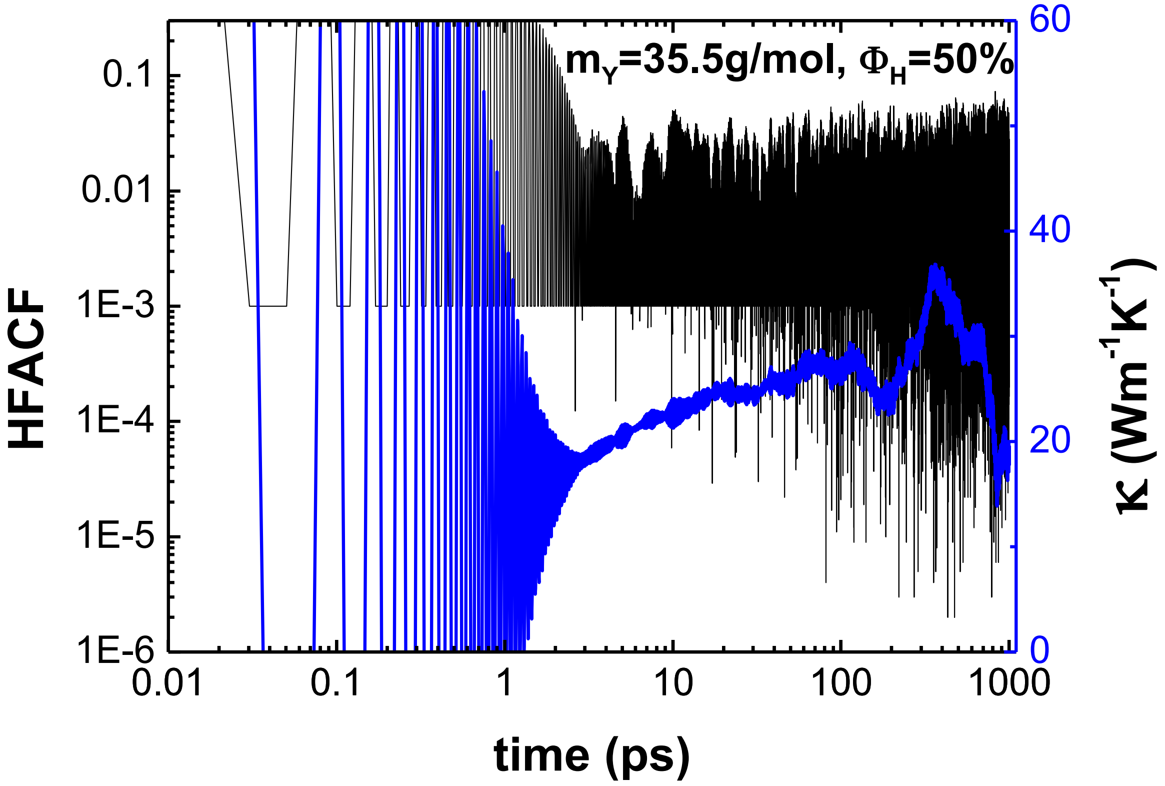

Figure S4. The heat flux autocorrelation function and thermal conductivity versus correlation time for Model C-H_mY_n with m_Y = 35.5 g/mol and Φ_H = 50%.
